# Reducing global health inequalities for a rare disorder: evaluating the international Prader–Willi Syndrome Organisation’s Echo^®^ programme

**DOI:** 10.1186/s13023-022-02504-5

**Published:** 2022-10-21

**Authors:** Tanzil Rujeedawa, Nora McNairney, Shelly Cordner, James O’Brien, Georgina Loughnan, Anthony Holland

**Affiliations:** 1 University of Cambridge School of Clinical Medicine, Addenbrookes Hospital, Hills Road, Cambridge, CB2 0SP UK; 2 Department of Psychiatry Douglas House, 18b Trumpington Road, Cambridge, CB2 8AH UK; 3 International Prader–Willi Syndrome Association, Salisbury House, Station Road, Cambridge, CB1 2LA UK; 4 International Prader-Willi Syndrome Organisation, Churchill Fellow, Parrera, Australia; 5 Metabolism and Obesity Services, Royal Prince Alfred Hospital, Sydney, NSW Australia

**Keywords:** Prader–Willi syndrome, Rare disorder, Project ECHO

## Abstract

**Background:**

People with rare disorders face significant global health inequalities; the challenge is how to raise awareness and develop a nucleus of experts in a country who are then able to provide guidance to others in that country. The International Prader–Willi Syndrome Organisation (IPWSO) established Project ECHO^®^ with the aim of facilitating the sharing of knowledge and the building of international partnerships to reduce global health inequalities for a particular rare genetically-determined neurodevelopmental disorder, Prader–Willi Syndrome (PWS). Four different ECHO programmes were established for the following groups: (a) Individuals (usually parents) who had taken on a leadership role in their country; (b) health professionals interested in PWS; (c) professional care providers supporting children and adults with PWS; and (d) a Latin American ECHO in Spanish. The programme started in 2020 and an evaluation was undertaken after one year to determine: the extent to which IPWSO had been able to recruit and retain individuals globally; the nature and extent of any benefits gained from the sessions; and examples of how individual involvement in the programme had led to local benefits. The methods included analysing routinely kept process indicators and survey data from the attendees of one component of the programme (the Leadership ECHO), together with a qualitative analysis of survey data and recorded interviews of attendees from countries of differing socio-economic status.

**Results:**

We describe the IPWSO ECHO programme and report on the outcomes from the evaluation of one aspect of the programme, the Leadership ECHO. Attendance of the Leadership ECHO sessions was satisfactory, with a mean of 24.7 participants, with participants attending a mean of 5.67 sessions, i.e., 30% of sessions. There was also good global reach, with individuals attending from 34 countries, although there were notable geographic regions with very limited representation. Feedback and interviews demonstrated the positive impact of the programme with some early evidence of positive developments at national level.

**Conclusions:**

Families and professionals from countries with a range of expertise and services offered to people with PWS remained engaged throughout the ECHO programme, established networks of support and fostered the development of good practice.

## Introduction

It is estimated that there are over 7000 rare diseases and disorders, affecting between 5 and 8% of the population [1]. The extent of knowledge and access to treatments varies significantly across the world, and rare disorders can remain undiagnosed or misdiagnosed for long periods of time [2]. In 2019, with the aim of reducing such global inequalities for people with one rare neurodevelopmental disorder, Prader–Willi Syndrome (PWS), the International Prader Willi Syndrome Organisation (IPWSO) developed their ECHO programme (ECHO: Extension for Community Healthcare Outcomes), based on that first established by the University of New Mexico. ECHO projects have been found to increase knowledge, improve health care in deprived areas [3], and reduce regional disparities in care [4]. In this paper, we describe the ECHO programme developed by IPWSO, report on an evaluation of the one part of the programme and consider its potential for other rare disorders.

The IPWSO Project ECHO programme, like all such programmes, uses internet-based technology to share best practices and undertake case-based learning with regular outcome monitoring to enable the regular evaluation of the programme. By making use of videoconferencing technology to link groups from different geographic areas, such programmes support the sharing of knowledge and enable communities to be equipped “with the right knowledge, at the right place, at the right time”. Project ECHO is based on the concept “all teach-all learn” with everyone sharing their experience and knowledge, with a resultant amplifying effect as expertise and knowledge spreads to where it was previously absent.

### Prader–Willi syndrome

Prader Willi Syndrome (PWS) is a rare and complex genetically determined neurodevelopmental disorder affecting all genders and races equally. It occurs in about 10.7 in 100,000 live births. The two main causes are the presence of a chromosomal deletion of paternal origin at 15q11-13 or a chromosome 15 maternal uniparental disomy. A third cause accounting for less than 5% of cases is a defect of the imprinting centre at that locus. The typical presentation is one of extreme hypotonia and failure to thrive at birth; followed in early childhood by the development of severe hyperphagia, which leads to life-threatening obesity unless access to food is controlled; the presence of developmental delay and intellectual and cognitive disabilities; relative growth and sex hormone deficiencies of hypothalamic origin [5] and a particular neuropsychiatric phenotype. An early genetic diagnosis, information for parents, and access to various interventions, including dietetic support, physiotherapy, speech and language therapy, and growth hormone supplementation are important in early life and a multidisciplinary approach has been shown to improve both the quality of life and health of those affected.

### The challenges for rare disorders

The Economist [6] highlighted the disparities experienced specifically by people with rare neurological diseases/disorders, such as PWS; even if the diagnosis is suspected, clinicians may not have access to the laboratory resources and the multidisciplinary clinical expertise necessary for diagnosis and treatment [7]. In addition, the geographical distance to where the expertise is based may be significant and access to the necessary funds or ability of the family to take time off work may be problematic, leading to an urban/rural divide and to additional health inequities within countries [1]. The International Rare Diseases Research Consortium (IRDiRC) was created to address these inequalities observed particularly with rare disorders [8, 9].

It is within this global rare disease environment that IPWSO [10] operates, seeking to both act as an umbrella organisation for national PWS Associations and also supporting people with PWS, their families, and professionals in the majority of countries in the world where there is limited knowledge about or support available for people with PWS. In these countries the aim is to act as the catalyst for the development of centres of expertise, which then train health and social care professionals. We describe the IPWSO ECHO programme in general and report on the evaluation, focussing specifically on one part of the programme—the Leadership ECHO (described below). The other ECHO programmes were still in progress at the time of this evaluation. The evaluation had the following aims: (a) to determine whether the Leadership ECHO had been able to identify and retain key personnel from different countries; (b) the extent of their satisfaction with the programme; and (c) to establish whether the stated aim of the Leadership ECHO of stimulating more substantial developments and networking was also achieved.

## Methods

For context, we briefly describe the development of the IPWSO ECHO programme. The evaluation used a mixed-methods approach. All participants gave their consent to the collection of quantitive data at the time of registration for the programme. Participants were also asked to enter their names in the chat box to confirm their attendance. Data included the use of routinely kept process indicators such as registrations, information on the regularity of individual attendance and the global spread of the attendees taking part in the ECHO programme as a whole and in the Leadership ECHO specifically, together with survey data from the Leadership ECHO attendees and a qualitative analysis of semi-structured interview data collected from a sample of attendees who represented countries of differing socio-economic circumstances. The interviews with selected participants were recorded and one of the authors (TR) undertook a thematic analysis. The selection and characterisation of specific themes was undertaken as part of regular supervision. The University of Cambridge Psychology Ethics Committee gave ethical approval for the evaluation (reference PRE.2021.060).

## Results

### Developing the IPWSO ECHO programme

IPWSO Trustees attended the ECHO programme at the ECHO Institute, and in subsequent consultations identified three key roles that an ECHO programme might fulfil: first, to support individuals (usually parents of children with PWS) or small interdisciplinary groups based in specific countries to take a leadership role in their countries; second, to provide a regular virtual environment for key health professionals in that country to develop their competency at assessment and treatment of people with PWS; and third, to provide a regular virtual environment to support the development of expert social care support in the different countries. To meet the needs of different stakeholders and to foster the development of expertise IPWSO established different ECHO programmes, which were implemented in stages. These included: (a) a Leadership ECHO, (b) a Health ECHO, (c) Professional Caregivers ECHO; and (d) Latin American ECHO (in Spanish). Whilst each of these ECHOs have a different focus and participants, the basic format for each is the same, with regular meetings by Zoom varying from weekly to monthly, and each session including a 20 min formal presentation, group discussions, and the presentation of challenges or cases from different countries.

The ECHO programme was administered by IPWSO and, following a pilot programme, it was then funded by an educational grant from Pfizer. The funders had no say in the programme or content. A small IPWSO ECHO advisory group, which was chaired by the IPWSO Vice-President, was established. At the beginning of the programme administrative support for the pilot was provided by existing IPWSO staff. When the above grant was awarded additional support was available. The Leadership programme was promoted through National PWS Associations and known contacts in countries with no PWS Association. It was also advertised using social media and by word of mouth. Later with the extension of the ECHO programme to other groups the wider ECHO programme was promoted through clinical and social care contacts in different countries and through the established Latin American PWS network.

For the Leadership ECHO specifically the focus was on developing the expertise and services in different countries with respect to their specific needs. Initial topics for discussion were determined by the IPWSO ECHO advisory group and later by the ECHO group itself. An expert was then asked to present on the chosen topic (see https://ipwso.org/how-we-can-help/project-echo/about-ipwso-leadership-echo/ipwso-leadership-echo-resources/). Topics of presentations included the following: establishing and maintaining a National PWS Association, managing emergencies, clinical trials, ethics or care, sibling experience etc. with presentations from parents and professionals from a variety of countries. At each ECHO session, in addition to the topic presentation, volunteers were asked to present a challenge or case at the next session. These challenges were often related to the earlier presentation and were illustrative of particular issues in that country. Prior to the session those presenting the challenge were contacted and supported with the development of their presentation. Challenges presented may not be concerned with a particular individual or family issue but, for example, with services or models of care in that country. The aim was to use the challenge sessions as a focus for debate and to encourage ideas and experiences to be shared. Often the group broke into smaller on-line discussion groups before coming together for the larger group discussions. Any presentations were made available on the IPWSO website.

**Fig. 1:**
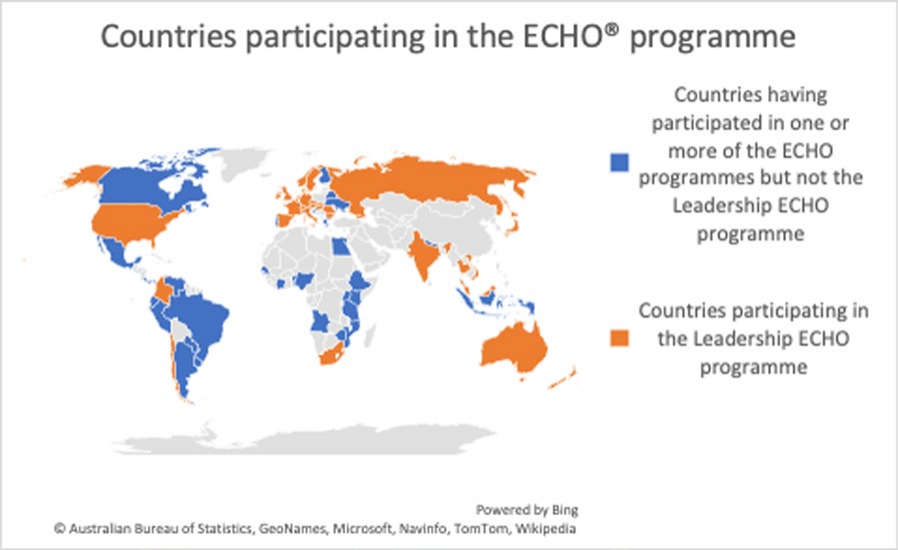
Countries participating in the Echo^®^ programme

We describe the overall programme and then focus specifically on the Leadership ECHO (Table 1 and Fig. 1).

**Table 1:** Data from the IPWSO ECHO programme

	Pilot leadership ECHO	Leadership ECHO	Health ECHO	Latin America ECHO	Caregivers ECHO
Launch date	28 April 2020	1 December 2020	16 February 2021	17 March 2021	12 May 2021
Number of meetings completed	9	10	6	17	3
Format/number of sessions in programme	9 × 90 min sessions	10 × 90 min sessions	10 × 90 min sessions	17 × 3 h sessions	12 × 90 min sessions
Number of didactics	10 by 9 individuals	13 by 12 individuals	6 by 6 individuals	24 by 22 individuals	3
Number of challenges/case studies	14	11	5	30	3
Number of countries participating	25	34	44	13	30
Delegates registering	55	80	134	430	148
Individual attendees	48	74	60	217	
Mean number of participants per session	21.9	27.6	25.5	92.8	
Time zones	Western Europe–New Zealand	Western Europe–New Zealand	Western Europe–New Zealand	Latin America	East Coast USA–New Zealand
Audience	PWS member associations	Leaders of PWS associations and parents—worldwide	Multidisciplinary/health and allied professionals—worldwide	Spanish speaking Multidisciplinary/health and allied professionals	Caregivers and providers

A summary of the iECHO data for the different ECHO programmes is given in Table 1. In total individuals from 34 different countries attended (see Fig. 1)

### Quantitative analysis of data from the leadership ECHO

**Fig. 2:**
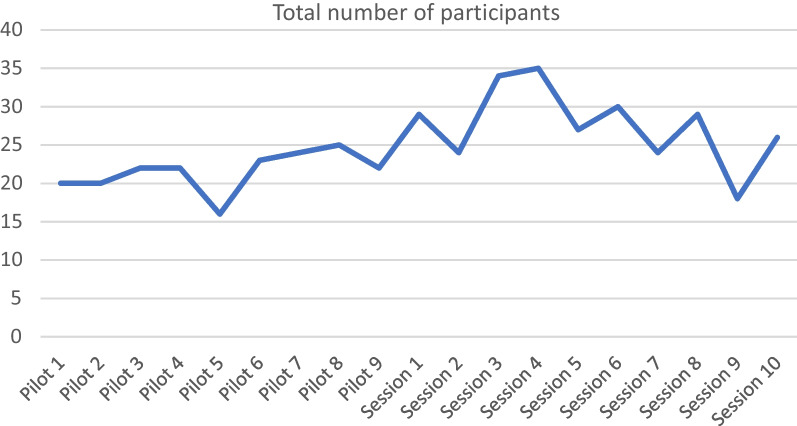
Number of participants attending the Leadership ECHO over time

The mean number of participants per session was 24.7, including pilot sessions, and the mean number of sessions attended per participant was 5.67, (30% of sessions), including pilot sessions. Figure 2 shows the number of participants plotted against time, indicating a consistent level of attendance. According to the ECHO Institute the ideal number of participants for optimal retention and engagement is between 20 and 30. This is in line with the number of participants in the Leadership ECHOs.

Participants from 34 countries registered, 97% (33/34) attended at least one session, and attendees from 18 countries presented challenges during the sessions. The didactics presented were recorded and made accessible via the IPWSO website and YouTube. Of the 6 h of video content available the mean number of views per Leadership ECHO video on YouTube was 14.19 (as measured on 3 August 2021). A poll conducted during the tenth session showed that 88% of the 26 participants had accessed the ECHO resources within the last month.

### Analysis of feedbacks and surveys

**Fig. 3:**
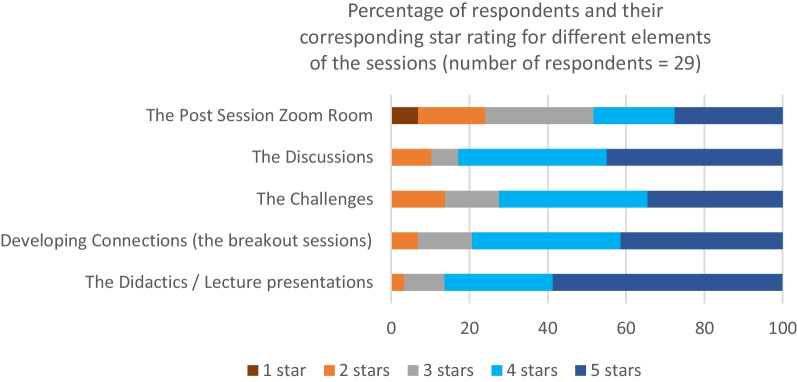
Graph showing how helpful the different parts of the sessions were rated

An average of 49.8% of participants responded to anonymised online surveys after each session, and 69% (29 out of 42 people) of those who had attended three or more sessions responded to a survey at the end of the Leadership programme. Feedback was overwhelmingly positive. Of note is that the statement “I am satisfied that the Leadership ECHO programme met my needs and expectations.” received a mean rating of 4.5 out of 5. Figure 3 shows the main components of each Leadership ECHO session and the corresponding star rating given by the respondents of the survey.

Analysis of the comments in the feedback forms identified three broad themes.

#### Theme 1: cultural and global issues

The global nature of the ECHO programme highlighted the following problems: (a) different countries are at very different stages of development and have different needs and priorities; and (b) time zone differences meant that some participants had practical difficulties in attending sessions. When asked if there were any models of good practice in their country for supporting people with PWS, the answers were overwhelmingly negative and these sessions provided both inspiration and a source of knowledge. However, as illustrated by the quote below, cultural differences meant that, for example, support models from elsewhere in the world may not be applicable.

*…in this country we still live with or live near our parents as extended family.*

#### Theme 2: impact of the leadership ECHO sessions on participants

Participants described being more confident talking about PWS with medical professionals, family, friends, and the wider communities. They were also more confident in acting as a resource for other people in their community, thereby strengthening their national PWS Association or the network of families in their country.

*I feel more confident that I can help parents based on the large amount of resources available to me through IPWSO and the other Associations that I have met that have offered assistance.*

The sessions were also reported to improve networking within the PWS community thereby decreasing isolation. In the survey, the following statements “I feel comfortable contacting the Leadership ECHO participants for support and guidance.” and “I feel more connected to the wider PWS community.” received a mean rating of 4.5. In addition, an overwhelming majority agreed that the sessions helped in forming new connections, which they highly valued. The sessions gave individuals ideas on how they and their associations were doing, with newer associations learning from more developed ones and implementing changes.

*Providing insight into how PWS is handled in other countries as well as providing the latest up to date on treatments. It provides a fantastic opportunity to build a network around the world. The exchange of ideas and the opportunity to ask for support (and to get feedback and help) is priceless.*

*I got to know fantastic people, parents from all over the world. Our National Association makes the first steps to build up its strategy, its structure, and I’m learning a lot from the more experienced organizations every time. I see the situation much more comprehensively.*

According to survey data, participants understood more about PWS; how people with PWS feel and think; and current health and social care recommendations and best practice. In addition, 15 of 16 participants agreed that the sessions were a help in their leadership roles. There was also evidence that the knowledge learnt during the sessions was further shared within regional communities, and several participants were keen to establish ECHOs within their countries.

*Based on the experience from the Leadership ECHO we have now set up monthly video calls in our country to connect our PWS community on a regular basis. Topics from the ECHO meetings have been used to communicate the latest status on care and treatments. So there has been a direct impact on the spread of information about PWS to our country members due to the ECHO Leadership programme.*

#### Theme 3: real world impact of the sessions

Whilst the evaluation did not directly assess the effects of the sessions on people with PWS, most participants (14/16) agreed that the sessions had helped them to think of ways to implement changes in their communities such as how to help teachers in their countries better understand the child with PWS. The sessions had also provided them with specific methods to be used to manage problem behaviours, which can severely impact learning in schools. Some participants described plans to connect with police and schools to improve their information about PWS and to create an information sheet for emergency incidents. One session gave participants ideas on how to use social media to spread good practice, a strategy that most seemed to have ignored until then.

### Qualitative analysis of interviews

At the end of the first series of Leadership ECHO sessions participants were selected via purposive sampling for a detailed interview. A total of eight interviews were conducted with participants from countries of differing socio-economic status. The interviews demonstrated the overall satisfaction of the participants. Although themes similar to those considered above were noted, such as improved confidence, examples of benefits described in the interviews are considered below.

#### Theme 1: how the ECHO sessions helped

All the interviewees viewed the ECHO sessions as very helpful and they had passed down the information learnt during the sessions to their communities, thereby promoting awareness. In one country, they had devised brochures educating the police and staff in schools and hospitals about PWS. One of the interviewees described that the biggest impact that the ECHO sessions had was having a network of people with whom they could share their problems and find solutions. They were now connected to knowledgeable people who could answer their questions, and questions that were passed on to them by members of their communities. There was a sense that an interconnected network of people had formed even outside the ECHO sessions. This has in fact contributed to the creation of the Asia Pacific PWS Seminar. In addition, one of the less developed associations has collaborated with a more developed one to establish materials to “reach and teach the teachers”. Two PWS associations are collaborating to encourage inclusivity and the active participation of people with PWS in the decisions that affect their lives. This initiative has already started pilot sessions and is aiming to improve the confidence of those with PWS. Meeting other people in similar situations also provided motivation for some interviewees to keep going and not give up.

Moreover, in one instance, an interviewee tried to implement virtual clinics, although to no avail, due to the lack of interest, at present, from medical professionals. Another interviewee is trying to get their government to acknowledge and uphold the rights of people with PWS, while also acknowledging that due to their condition, they have specific needs. Another is aiming to set up a home for adults with PWS in their country. Motivation for pressuring governments for better PWS care has also been a recurrent theme. One interviewee has managed to get their government to consider the provision of GH therapy at subsidised cost for people with PWS. Use of social media has also been implemented by several of the interviewees.

#### Theme 2: country specific problems

An important issue highlighted during the interviews was the uniqueness of each country. Interviewees highlighted that, while a lot of the advice and guidance from the more developed associations, usually in high-income countries, was relevant to them, not all of it was. There were socioeconomic, geographical and cultural differences. A particular issue highlighted by several interviewees was the lack of government funding and some of the advice given required proper funding. Additionally, in some countries, on-going support by the extended families is the expectation and as such, care homes were rare. Language and the quality of internet access are also issues that have prevented some of the members from sharing information. Despite the differences between countries, many of the interviewees pointed out that the everyday problems of children and adults with PWS and their families are broadly similar, and as such, the sessions were still very helpful.

#### Theme 3: ideas about the future

Interviewees were very satisfied with the ECHO sessions and were very keen to give feedback on how to improve the sessions and the work done by IPWSO. One of the interviewees wanted more standardised information, having found the standardized clinical guidance provided by IPWSO very helpful. They believed more information would be useful, notably regarding diets. Providing more opportunities for networking was also a recurring topic, as was the provision of more assistance and grants to the poorer associations.

## Discussion

The aim of reducing global health disparities with respect to rare neurodevelopmental disorders, such as PWS, presents with particular challenges, including raising awareness of the disorder, having the clinical and laboratory expertise to arrive at an accurate diagnosis, and finally ensuring that patients and their families have access to the multidisciplinary expertise and knowledge they require for informed life-long support and treatment. This evaluation has shown that Project ECHO, specifically the Leadership ECHO, had been able to engage with individuals in different nations; maintain an adequate number of attendees and their involvement over time; facilitate links between individuals and countries, thereby reducing isolation; and had given individuals the confidence and knowledge to consider ways of improving the lives of people with PWS in their countries. Although the evaluation did not determine directly whether change took place, the feedback did demonstrate changes in behavioural intent that have been shown to lead to changes in behaviour [10].

The IPWSO ECHO programme had the wider aims of not only providing a forum that would encourage local development in knowledge and services (Leadership ECHO), but also providing a safe and informed environment for health and social care professionals to share ideas, discuss challenges, and support each other (Health and Professional Care Providers ECHOs). With respect to the Leadership ECHO in particular, there were concerns about the extent of its global reach with the attendees being predominately from Europe. Two additional concerns were: first, the need for cultural awareness and the recognition of specific local circumstances, whether financial or cultural; and second, how to encourage attendees to aspire for the best but also to recognize the differing realities around the world.

The challenge, particularly for a small global charity such as IPWSO, is to accommodate differences in need between countries and also to appreciate and address the cultural differences, such as those listed above. This evaluation highlighted the importance of IPWSO having a diverse and globally spread Board of Trustees and advisors. In response it is of note that membership of the new Board of Trustees covers North and South America, Europe, and the Asia Pacific Rim. However, there remain significant global gaps, particularly in the African continent though IPWSO has recently co-opted a parent to the IPWSO Board from South Africa. There is also now a psychologist and geneticist from South Africa and Kenya, respectively, as advisors to IPWSO. IPWSO has also linked with African rare disease organisations. The IPWSO Board of Trustees is also considering establishing global groupings based around geography and/or a common language. An example of this is the Latin American PWS network, which has hosted an ECHO in Spanish.

The IPWSO ECHO programme is still relatively new, and it was recognized from the beginning that change in some countries will take a long time and that it will be an iterative process. The running of the IPWSO programme is demanding, and to make the most of the materials requires access to high quality administrative support. This evaluation indicates that there is a need and that people from many countries wish to engage in such a global programme. A small international rare disorder charity, such as IPWSO, with internationally recognized clinical and scientific expertise, together with parents, is in a unique position to undertake such an initiative. Central to any such initiative is an understanding that information made publicly available through didactics and the IPWSO website must be based on the best science, enriched by the shared experiences of all who take part in it.

## Conclusion

This evaluation of the IPWSO ECHO programme has shown that parents, clinicians, and others concerned with the care and treatment of people with PWS in different countries were willing to engage in the ECHO programme and they maintained their engagement over time. The feedback after sessions was overwhelmingly positive. The benefits include the sharing of ideas, making contacts with other individuals and organisations, and developing a community of support. There was also some early evidence that this led to changes in countries that may then bring benefit to people with PWS. Whilst there was a good global reach there were areas of the world unrepresented and efforts need to be made to engage with potential leaders in those countries. There were examples of some cultural differences in the support of people with PWS and those who were facilitating sessions needed to be aware of them. However, we see these differences as strengths of such a global programme potentially challenging established thinking. Time zone differences and ensuring ready access to the internet gave rise to practical challenges.

## Data Availability

The data is kept by the International PWS Organisation and requests for access should be submitted to one of the authors (SC) at IPWSO.

## Abbreviations

ECHO: Extension for community healthcare outcomes
IPWSO: International Prader–Willi syndrome organisation
PWS: Prader–Willi syndrome
